# Brave Clarice—healthcare serial killers, patterns, motives, and solutions

**DOI:** 10.1007/s12024-022-00556-4

**Published:** 2022-12-01

**Authors:** Rahma Menshawey, Esraa Menshawey

**Affiliations:** Kasr al Ainy Faculty of Medicine, Cairo University, Geziret Elroda, 11562 Manial, Cairo, Egypt

**Keywords:** Serial murder, Healthcare, Homicide, Murder, Forensic science, Law, Ethics

## Abstract

Healthcare serial killing involves the intentional killing of multiple patients by a healthcare professional. It is a formidable challenge to identify in the medical context, and a daunting legal task to prove beyond reasonable doubt. What can be done or remains to be done to intercept these serial killing events and help serve justice, while at the same time not risk dismantling public trust in the healthcare system? In light of several recent modern charges of murder against healthcare practitioners across the world, this review aims to report the themes, patterns, and motives of medical serial killers as well as highlight areas of work on both medical and legal fronts to help identify these events, and to most importantly protect the vulnerable patient community.

## Background

Healthcare serial killing involves the intentional killing of multiple patients by a healthcare professional. Ultimately, the practitioner-patient relationship is a trust-based one, where the patient places trust in doctors, nurses, and the healthcare system [1]. This opens a vulnerability to those with ulterior motives.

Healthcare practitioners have intimate knowledge of the process of human life and death. This, paired with the facilities that hospitals provide and the tools such as paralytics and lethal doses of medications, as well as the general anticipation of death as a patient outcome (thus garnering little suspicion), maybe the attractants of those opportunistic killers.

In this explorative review, we analyze in depth the topic of the healthcare serial killer and summarize the commonalities and themes among cases in the literature, as well as the challenges faced across both healthcare and legal systems, and the potential safeguards that can be implemented across several systems to protect the patient community. The insights derived in this review are scoped from a variety of cases and studies in the literature from different countries and systems.

Medical murder is a two-pronged modern problem that requires simultaneous and synergistic efforts from the medical and legal perspectives, as well as swift interception in order not to weaken the trust between the general public and their healthcare systems.

## Main text

### Homicide and serial killers—criminology perspective

“I controlled other people’s lives, whether they lived or died. I had that power to control. After I didn’t get caught for the first fifteen, I thought it was my right. I appointed myself judge, prosecutor and jury. So I played God,” Donald Harvey, hospital orderly, killed 87.

Homicide is the killing of another human being which may be divided into murder, or accidental (manslaughter), lawful with a defense (i.e., insanity, or self-defense) also known as justifiable homicide, assassination (killing of a recognized person), euthanasia, and as part of war crimes. Murder is considered an extremely serious crime by most societies, and may be punished with life in prison, or capital punishment (state-sanctioned killing of a person) [2].

Holmes and DeBurger devised the main typologies of serial murder including visionary (hallucinations based), mission oriented (killing those “lesser” than), hedonistic (sensational killers), and power-control (domination over victim seekers) [3].

This typology maybe seen as an organized-disorganized spectrum of killers, with visionary on the disorganized end and power-control type on the organized end [4].

A serial killer is conventionally defined as one who murders multiple people—though there is lack of consensus on the exact definition [3, 5], and this has stifled meaningful quantitative research in this area (jurisdictions vary in the number of killings from 2, 3, or even 4 in defining serial murder). According to psychopathology, a serial killer is exceptionally skilled at impression management, acting above suspicion with a persona of innocence used to lure and conn their victim and deflect suspicion (social blending) [4].

In the realm of empirical research, a robust study on cases of serial killers revealed that the majority of solo serial homicide offenders are male (with serial killing being defined as the act of 2 or more discretely separate killings) [5]. The majority of serial killers were found to be in the Americas compared with other continents.

The perpetrator-focused interest in the literature has left victim data and details limited, and this is an area of future work and research, as a paradigm shift is needed with a victim-centered focus in order to prioritize vulnerable peoples.

### Psychopathology of homicide

There are two main defining psychological characteristics of a serial killer which are compartmentalization and dehumanization. To neutralize feelings of guilt, serial killers compartmentalize. This allows them to develop 2 distinct social circles: (1) a close circle of friends or family whom they care about and typically do not harm, and (2) a victim group—strangers to whom they have no remorse [4].

A modern example includes Nazi doctors who used “doubling” to create 2 distinct selves—one that engaged in experimentation and extermination of inmates, and another that maintained their life outside of concentration camps [6].

Physicians are suggested to be more susceptible to doubling than any other profession. The medical profession requires one to act objectively and mundanely in the presence of blood, trauma, and corpses. This allows for desensitization to death and an ability to maintain highly skillful cognitive function under circumstances that may otherwise be deemed abhorrent or reprehensible in the eyes of lay people [4]. The physician therefore develops a “medical self.” In the case of prolific medical serial killer Dr. Michael Swango, who killed up to 60 patients under his care, he described in his diary how the “sweet husky close smell of indoor homicide” served as a reminder that he was “still alive” [4] (see Table 1).

**Table 1:**
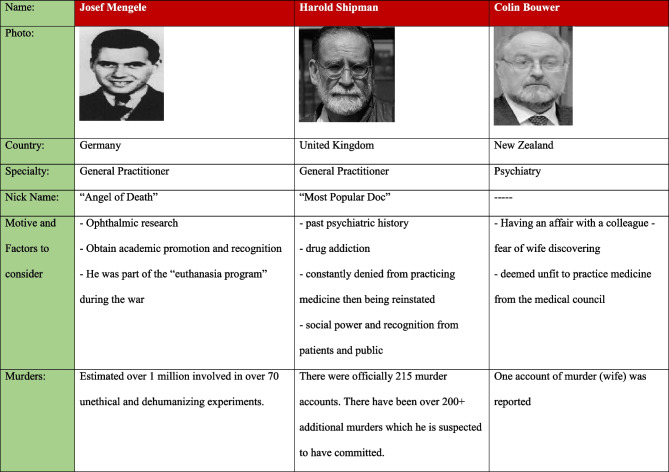

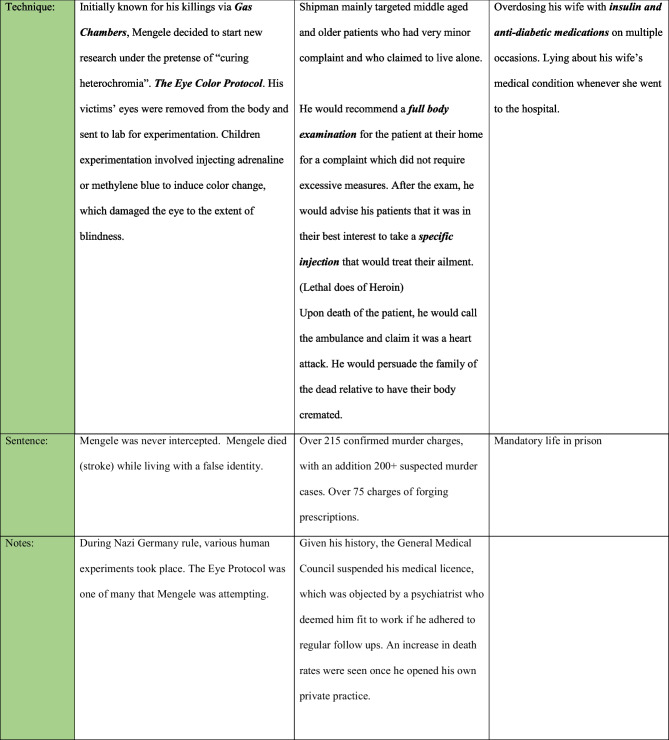
Characteristics of three medical serial killers—Mengele, Shipman, and Bouwer

### In addressing medical malpractice, are we missing medical murder?

Medical malpractice may lead to the death of a patient in cases of such reckless endangerment of human life, lack of providing adequate care, and failure to prevent antecedent error. Trends have shown an increase in police reports by physicians and next of kin regarding the death of a patient with consequent increase in criminal prosecution, in the past decade throughout the world [7].

Laws however are variable worldwide on the degree of culpability of gross negligent manslaughter that may ensue from substandard care. What exists is a scale of negligence where on one end, doctors are almost *never* held criminally responsible (only 1 doctor has been criminally charged in New Zealand in a 10-year period) to another end including English Law where extreme negligence itself is sufficient to prove *mens rea* and is criminally prosecuted [8].

These variations in tests and interpretations may either be (1) setting the bar too low for what is criminally culpable behavior or (2) setting the bar too high with little consideration to other factors of reasonableness or culpability leading to increased criminal prosecutions that may be counterproductive. The use of defensive medicine is an example of this [9]. Ultimately, the Law holds the right to address reprehensible behavior when it leads to a criminal act [8, 10]. To quote the words of Dr. Michael J Powers QC:

“…society has the right to control bad professional care…whether or not a doctor is going to find himself on a manslaughter charge will really depend on how much thought, how much care and how much attention he is giving to what he does…The cases where we have seen convictions are cases which stick out like a barn door” [11]

In the midst of these arguments and hypotheticals of culpability, and scales of negligence, the medical serial killer remains poorly addressed. Perhaps, it is out of the Laws intent on *fairness* on when to hold someone criminally liable that has led to much delay and the prosecution of only those glaringly reckless cases that “stick out like a barn door,” as well as the medical community’s personal bias that prevents them from appreciating that such an opportunistic killer *can even exist* in their realm. This is evidenced by the glaring “paucity of the medical literature” on this topic, as well as the inability to make amends with an example being the circulation of tissue and skeletal remains of Nazi Germany Jewish inmates in German Medical school’s well into the year 1989 [12].

One aspect is evident, that there exists a subtype of homicidal persons who are healthcare practitioners and conduct themselves within the current framework of healthcare systems and patient relations. We may consider that the healthcare serial killer is a distinct enough category of serial killers. This is evidenced by their decade’s long careers and late apprehension—*if ever*—after tens and hundreds of victims (and millions if we count those doctors whose murders are backed by a prevailing political ideology). The medical serial killer is ultimately considered one of the most difficult perpetrators to bring to criminal justice [13].

### A brief history of medical murder—modern and past cases

“They are woven throughout medical history—these grotesque practitioners—poisoners, smotherers, pitiless experimenters, and, most alarming, those whose crimes are still undiscovered, whose names are still unknown.” [14]

Some would suggest that clinicide (the unnatural death of patients under the care of a healthcare practitioner) is a relatively recent phenomenon; however, it remains difficult to place along the timeline of human existence given several factors—such as the difficulty of identifying when these events are occurring. It is believed that medicine has offered up more serial killers than any other profession, second only to nursing [15]. Healthcare serial killers may encompass a variety of healthcare professionals (i.e., physicians, nurses, dentists, pharmacists, hospital orderly), and may include varying settings such as the hospital, nursing homes, or the outpatient clinic. Overall, cases of serial healthcare murders are rare; however, they are largely under-represented due to issues with detection as evidenced by killings that may span decades long into a practitioner’s career [16].

More glaring examples stand out in the form of horrific human experimentations and murders of inmates under Nazi Germany [17]. In fact, in Nazi Germany, more than half of all of Germany’s physicians were enrolled within, and with their expertise and scientific background did the Nazi medical program take its full form in the shape of the “medicalization” of horrific concepts of eugenics, sterilization, and ultimately genocide [17]. The participation of German physicians was an early enrollment, self-elected, and safe to say—unanimous.

Current modern examples can be seen in the USA (Ohio) with 14 charges of first-degree murder against Dr. William Husel for utilizing significantly excessive and lethal doses of fentanyl—where prosecutors were stressing the unusual doses as high as 2500 µg as a clue to an intent to end lives [18, 19] (found not guilty) [20]. A jury found a California doctor (“Lisa” Tseng) guilty of second-degree murder by overdosing her patients with controlled substances for financial gain—the first case of its kind in the USA where a successful guilty verdict was given for the overprescribing of drugs [20].

The murder of patients may be associated with a sexual component, such as a sexual assault before the murder, or sexual gratification afterwards, an example being Dr. Ronald E Clark in the 1970s who sexually assaulted his patients then murdered them using sodium pentothal [15].

Some serial killer doctors indulge in mercy killings involving chronically ill elderly persons where they may claim they wished to ease the passage of their patient into death, an example being Dr. John Bodkin Addams when in 1957 he admitted to killing more than 400 elderly women (he was also included in 132 of their wills, and a jury found him not guilty) [15].

It might be wise not to assume that healthcare serial killers constitute isolated events in history as increased awareness in modern times has resulted in open investigations and even exhumations for missed homicides, which suggests that it is a relevant problem [21].

### Types of medical murderers

FBI criminal profiler Peter Smerick distinguishes between two types of medical killers: (1) the mercy killer who rationalizes that they may murder the patient to end their suffering and does so relying on the fact that autopsies are rarely done on patients who are critically ill, and (2) the hero killer who puts their patient at a substantial risk, and if they succeed, they will be a hailed as a “hero” [22].

Doctors as a group show some of the highest rates of murder compared to any other profession [23].

Kaplan describes the categories of medical killers as follows:

1. *Medical serial killers*—these are doctors who derive a perverse pleasure from killing their patients. The number of victims is numerous and may exceed the hundreds or thousands (substantially higher victim counts than non-medical serial killers), examples being Shipman and Swango who killed between them more than 300 patients.
2. *Treatment serial killers*—these are doctors who take on risky treatment options for their patients with the awareness that their action is leading to the demise of their patients, paired with a refusal to desist their treatment plan or acknowledge the risks involved [22].
3. *Political mass murders*—these are accomplices in genocide or brutality with the support of their government. One example is that of Dr. Joseph Mengele who during Nazi Germany enacted horrific human experimentations on inmates (see Table 1) [24, 25]. The pivotal role of doctors in mass genocides is a curious phenomenon, and as Kaplan puts its “medicine contains the seeds of its own destruction is confirmed by the recurrent involvement of doctors in genocide” [22].

### The motives

“Throughout history, they show recognized features of inflated sense of importance, and are often at the centre stage of praise within the medical community. The homicidal physician possesses an innate sense of drama coupled with unusual arrogance.” [14]

The motives behind these killings can be variable, or maybe totally unknown (see Table 2).

**Table 2:** Killers versus victims

**The killers**	**The victims**
• Intensely narcissistic traits • If claiming to euthanize a patient to end their pain, closer scrutiny may reveal a secondary gain (financial) or motive of excitement and thrill in controlling another person’s life • Frequently performing tasks outside their ranks or hospital status • Frequently polarized opinions by coworkers • Extremely close to their supervisors which serves to provide protection from criticisms or reports—complaints are brushed off as mere professional jealousy • Men account for the majority of healthcare serial killers, among doctors and nurses alike • Lethal injections surprisingly can be done in the presence of family members who are unable to make the medical connection. This is a clue to the power-control themes • Falsification of credentials or achievements should be considered a *high-risk factor*, even in the lack of a previous criminal history	• Selection of victims is very important and specific—the very old and very young • In the rare scenario that a victim can recognize the situation and can complain, their complaint is ignored • In cases where the patient explicitly mentions they are going to be killed by a nurse or doctor to their family, this is often attributed to delirium, paranoia, or illness • Very old and sick patients with high morbidity, are likely victims as death maybe anticipated and little suspicion is considered

These motives can be *any* of the typical typologies [26], and the healthcare serial killer may adopt one or multiple of these motives. Attaining power and control over a patient or acclaim for successful diagnosis and saving of the patient are common themes. There is a particular scenario where the practitioner may be inducing cardiopulmonary arrests for the sake of setting off a code—this can be identified where there are frequent arrests followed by multiple successful resuscitations made by one caregiver. Here, the motive was thrill—to satisfy the “excitement” involved with reviving the patient, or to gain recognition and acclaim from their colleagues (like a fire-fighter setting a fire).

Financial gain is another common motive with collaboration with insurance companies and funeral homes having been reported. A clue to a financial motive may be noted if sudden alterations in the wills of the victim patient have taken place, allowing the practitioner killer to inherit their fortunes. Enacting a sexual fantasy or attracting the attention of a romantic interest is another potential motive [22]. A known example is the case of nurse Kirsten Gilbert where hospital policy demanded the presence of a security person whenever a code was called—the security person was her paramour [27] [28] (see Table 3).

**Table 3:** Two murdering nurses

**Kristen Gilbert, registered nurse, 1989, USA—life in prison**	**Genene Jones, vocational nurse, 1978, USA—159 years in prison**
A string of unexpected deaths on the floor where she takes her shifts Doctors observed this and some requested that she is not tasked to attend to their patients Deaths on her ward were 3 × more than other wards Became known as the “Angel of Death” Her period of occupation started in 1989 and the higher rates of deaths continued for years well into 1996 Coworkers’ suspicion caused one of them to monitor the number of epinephrine vials on the floor—3 vials were found in the resuscitation room and laced syringes in the trash	Early signs of bizarre and ritualistic behavior when pediatric patients died Was able to “tell” which patients were going to die on her shift Frustrated coworkers began to track statistical logs—inexplicable causes of deaths were identified The real alarm sign was not the number of cardiac arrests on the ward but rather the number of which where Genene was physically present Coworkers took these alarming statistics to their supervisors but were *ignored*; deaths continued to ensue One physician noticed recent coagulopathy events which he suspected were due to heparin. On questioning, Jones was asked what dose of heparin she was using, and her response was more than 1000 × the normal dose—hospital ordered all heparin injections to be witnessed by another person Jones took 1 month off work and during this time not a single code happened No evidence directly linking Jones to the deaths for one PRIMARY reason that plagues most health care serial killing events—the killer themselves writes the pre-arrest events. Upon her return from vacation, codes began again Chief of cardiovascular surgery had become unimpressed with hospital response, and threatens to refuse to send his patients to the PICU Emergency meeting was convened and included experts in the field from the USA and Canada—they concluded not enough evidence was found for homicide. They decided to restrict the nurses on that unit to registered nurses only which restricted Jones Jones quickly relocated to a friend’s clinic—a child at the clinic died under her care and trace evidence revealed a vial of succinylcholine that linked her to the crime. She was charged with 159 years

In some cases, the motives are purely sadistic where a preference by the killer was given to those patients that they deemed to be demanding or increased their workload. In the case of Orville Lynn Major, a nurse testified to the sudden collapse of her previously stable patients when under his care. Hospital pressure to free up beds maybe a motive to hasten the death of critically ill patients [27]. In this scenario, the patient is considered a “bed blocker” [29].

## Patterns to ponder

Some characteristics of the healthcare serial killer include a history of substance abuse, craving attention about his or her skills, a diagnosis of a personality disorder, a record of incidents at another hospital, and fabrication of credentials.

Characteristics that may be noticed by colleagues or staff members include suspicions or anxiousness when covering patients when they are on duty, noticing that the healthcare serial killer can always tell beforehand which patients are going to die and when (as well as inappropriate behavior like betting on which patient is going to die), and a higher incidence of death occurs when they are on shift (although statistics alone cannot prove guilt, they can help paint a general picture or initiate investigations). The healthcare serial killer may also have particular nicknames, i.e., Angel of Death [30].

A cluster of common themes in the serial murder of patients includes the following:

- Repeated cardiopulmonary arrests with a high rate of resuscitations
- Deaths that cluster around evening or night shifts

Yorker et al. have detailed the findings in 90 prosecutions involving caregiver-associated serial killing. Their findings suggest that the prosecutions were highest in areas with more advanced healthcare systems such as the USA and Germany [31]. The majority of those convicted were nurses. Gender wise, 49% of those convicted were female, and there appears to be a gender discrepancy involving male nurses which account for 6% of registered nurses but account for 44% of nurses prosecuted for murder. The vast majority of healthcare murders occur in hospital setting—and are not limited to only one area in the hospital (i.e., killing in the ICU and the outpatient clinic) [31]. It might be that countries like the USA and Germany have more cases of medical serial killing, as such advanced healthcare systems may have the means to intercept and address this problem.

A variety of methods may be employed by the killer with injections of insulin and potassium, or tampering with respiratory equipment (i.e., lowering the flow rate) being common choices [31]. Insulin injections will lead to hypoglycemia and coma and eventual death if not treated abruptly. These cases may be further compounded by a patient who is already diabetic where this malicious dose of insulin may be misattributed as an overdose by the patient themself. In a quarter of the cases, the method was unknown [31].

Toxicology is a staple in placing convictions and prosecuting cases of healthcare murders where poisons or paralytics were used [32]. Nonetheless, most of the calls to examine toxicology are post-mortem or post-exhumation where a high index of suspicion is present. This is a problem where rapidly degrading compounds are being used, an example being the use of potassium to induce cardiac arrest, where upon death, there is already an elevated level of potassium in the body [32, 33]. A high index of suspicion should be present where any substance is found to be elevated in a patient that *was not previously prescribed* to them and the presence of high concentrations of a substance in syringes, as well as fingerprint of the perpetrator on the syringe may all count as compelling evidence for serious medical error or a crime (see Table 4).

**Table 4:**
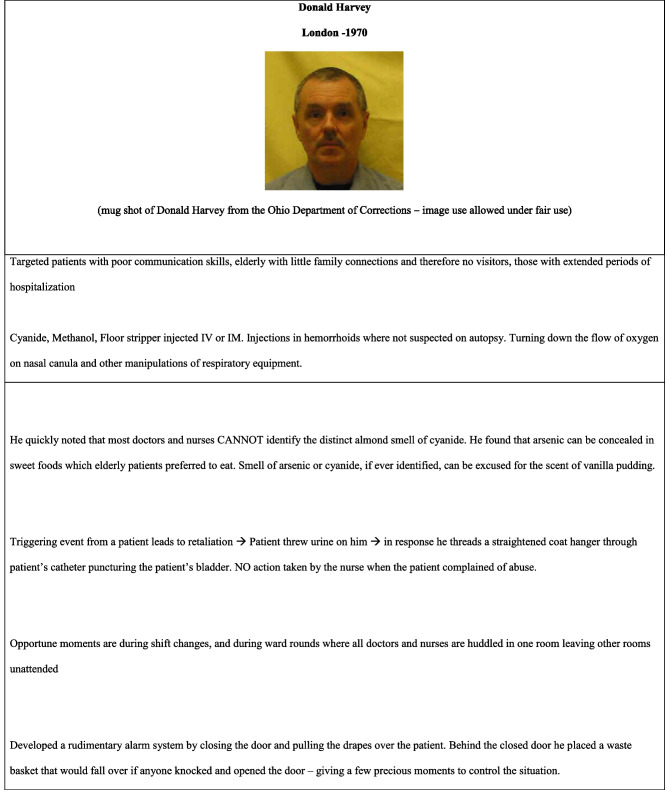
An unusual hospital orderly and his observations

Finally, the primary alarm call for any investigation typically comes from *coworkers* who have noticed unusual statistics revolving around the accused healthcare provider’s shifts or their direct eye-witness reports, rather than from concerns voiced by patient family or even medical examiners [25].

## What can be done?

Medical murder can be often missed—a point of recent focus in countries like Belgium and the Netherlands where modern trends in euthanasia policies have rung appropriate alarms for potential abuse [34, 35]. Furthermore, the true incidence of medical homicide “is impossible to determine,” as each healthcare system presents its own opportunities for malice. What is needed are the tools for early detection and confident (and potentially anonymous initial) reporting in order to prevent covert retaliation occurring to the reporter. All attempts to reconcile this issue with the Law and Public must also address the need to not undermine public trust in the healthcare system. This may be addressed by timely, honest, and transparent recognition of when suspicions arise.

Morbidity and Mortality (M&M) meetings are “a critical component of clinical governance” [36]. This is a meeting forum where patient deaths and poor outcomes are discussed in order to improve patient safety. In the USA, they are *mandatory* to attend. M&M meetings typically host an array of healthcare practitioners including doctors, nurses, medical students, and management. Ultimately, they provide the necessary assurances that poor patient outcomes are being addressed. These meetings may alert to any unusual outcomes or patterns and necessitate the need for disciplinary action.

One of the hindrances of such meetings is the many hurdles to open discussion, where one study detailed issues such as (1) an open investigation is occurring regarding the case, (2) fears of being judged for a mistake even though their specialty is associated with high mortality rates (such as emergency medicine), (3) meetings being used as personal vendettas, (4) meetings being rushed too soon after the event without enough relevant information or evidence, (5) presence of dominant personalities who would criticize or show off their own decisions, and (6) the presence of management/the desire for it to be a doctors only meeting [36, 37].

Overall, despite the threat of criminal prosecution, physicians are open to discussing adverse outcomes of their patients, but there remains considerable room for improvement in order to conduct these meetings in the most effective way possible [37]. These include keeping a goal-oriented approach to the meetings and a clear definition of outcome measures in order to determine and make a distinction between what may be deemed as a learning experience and what may progress into further investigations. Meetings should be properly timed when all the relevant information is ready, should be attended by a variety of persons, and perhaps should be delivered in a blinded fashion in order to avoid narcissistic displays by other doctors such as “criticism or showing off.” M&M meetings maybe a useful tool to triage cases of mortality and should be utilized in other healthcare systems worldwide and attended routinely [37, 38].

A systemic change is called for on healthcare systems to shift from denial of employment lawsuits, or wrongful discharge charges, to the absolute prioritization of patient safety, and recognizing that if a successful conviction is made, this will lead to more damaging wrongful death lawsuits.

There needs to be serious consideration of the intentional harm of a patient as part of training healthcare workers in patient safety. Hiring and screening processes need to be more robust—calls for background checks where there are gaps in employment history and questions into previous terminations should be made. Serious control over insulin supply is needed as it is a notorious drug of choice for perpetrators which draws little suspicion [16] (some countries have employed a 2-signature system needed for the prescription of potassium and insulin).

Evidence to the discrepancies and errors found in death certificates calls for standardization and appraisal of doctors’ abilities in this area [38, 39]. With regard to the exhumations, a recent attempt by the German Crown Prosecutor was conducted to identify missed homicides, where they found alarming differences between the true cause of death and that which was noted in the death certificate [38]. Thirty-nine out of the 155 exhumations were classified as possible medical malpractice with 7 death certificates labeled as totally erroneous causes of death [38].

The results of the exhumations have shed light on 2 primary issues. Firstly, if the initial death certificate indicated nothing worthy of suspicion by the doctor, then police investigation remained *superficial*, and no autopsy was ordered [38]. This is critical in that previous cases involving doctor serial killers have conducted their own death certificates to cover their crimes. It is also unusual that in the case of the exhumations, some of those suspected of medical malpractice showed extreme discordance with the true cause of death—even when the cause of death was *obvious*. The second point is that it was a “lucky coincidence” that inspired a re-evaluation of the case, typically another murder attempt by a suspect [38].

There appears to be a pattern of falsifications of documents or credentials—and these were often missed during hiring, and *if they were* identified, it *was not* a deterrent for hiring. Hospitals need to enforce stricter measures for determining the authenticity of the credentials of their workers and consider the practice of falsifying credentials as *a high-risk factor* for future criminal activity (even in cases of no previous criminal record) [40]. Countries such as the UK employ rigorous checks/certification process and revalidation which is aimed to promote a governed practice which can be used as an exemplar [41–43].

## Legal issues

A successful criminal prosecution in a case of healthcare serial killing relies on an array of evidence, including eyewitnesses, toxicology reports, confessions, and statements made by patient family and colleagues. A finding that a patient is more likely to die at the hands of an accused physician is insufficient to prove guilt of murder in a criminal court but may be exceptional in a civil suit. In the case of Micheal Beckelik, there was not enough evidence to prove guilt beyond reasonable doubt, but a civil suit charged him successfully for 27 million dollars [44].

The burden is on the prosecution to prove to 2 critical points: (1) the patient’s death was *unnatural*—and this can be a challenge given a predilection for victims of extremes of age or those with chronic illness and high morbidity—where death may be anticipated, and (2) that it is the actions of *this* particular healthcare provider that led to the patient’s demise [44]. Reasonableness is another challenge, as human error is deemed unreasonable, but in the context of a medical setting, the question becomes whether or not an action taken towards a patient would have been taken by a reasonable person under similar circumstances [44] with come consideration of the *ability to* provide a standard of care. Establishing the presence of implied malice maybe a determining factor in a murder charge versus a manslaughter charge against a practitioner.

Healthcare serial killers use a variety of defenses when intercepted and accused in court, the two most common being insanity and euthanasia defense. In the euthanasia defense, the killer may claim that they administered a lethal dose of medication at the request of the patient (the capacity of the patient and their explicit consent becomes the topic of argument in this case, as well as whether or not there are established laws or policies in place for physician-assisted dying, and if other palliative options were considered).

Hospital administrators are often uncooperative with prosecutors and may even obstruct the investigation with reasons being fear of negative publicity, civil suits, and poor record keeping. On the other hand, cases where the hospital was cooperative with *discreet* investigations resulted in the collection of evidence and successful convictions, and served as a deterrence for future suspicious activity [31].

According to the US Department of Justice, healthcare serial killing is a class of crimes considered to be the most difficult to detect and prosecute. Even in the rare scenario where it can be detected, proving guilt requires extensive medical knowledge and expert input [13].

With regard to the serial killer versus the treatment killer, we wish to highlight an important distinction. The treatment serial killer, whose arrogance and narcissism fueled by their lack of introspection or admittance of their own limitations, may typically be charged with manslaughter because a motive or intent remains unclear (if anything, it appears superficially that they are hopeful that their risky choice saves the patient life to increase their acclaim or reaffirm their self-confidence). The distinction with the serial killer remains in setting the precedent that there *is* a history of patients that are in fact treated well by them, within the guidelines of sound clinical reasoning and decision-making skills. If one can establish this, then the choice to resort to a different or deviant choice with another patient should be questioned. The treatment killer would have an all-around poor performance as a practitioner, with tales of their mishaps or negligence across the board. It should become quite unusual if one who is skilled enough to make the best decision opts instead under no particular stressor or change of setting, to make a poor decision that would lead to the patient’s demise. The decision to do harm in this exclusionary scenario would itself be the motive or the implied malice. This can only be done with an extremely thorough analysis and examination of all the patients that have ever entered their care—a task of determination that may set the foundations for which future healthcare serial killers may be addressed by the criminal law, and where charges of first-degree murder may be laid. To reiterate in making a case against a suspected medical *serial killer*, we recommend that one should not shy away from utilizing the fact that *there are* successfully treated patients by the accused of similar demographics or qualities or disease characteristics as those patients that are their victims. This makes sense in that running a successful practice serves the needs of the serial killer as this can build trust within the medical community, whereas tales of the treatment killers’ outcomes and negligence may precede them and even deter patients from them [45]. This observation maybe specialty specific—and may also explain the unusual longevity of the serial killer’s career as their “mishaps” or “outcomes” might never be addressed as anything more than that and may never see the light of a formal investigation.

## Conclusions

Healthcare professionals are already at a level above suspicion, as well as the advocacy of the medical community for a “no blame culture” towards addressing incidents where an adverse patient event may have taken place. Given the sheer number of victims of healthcare serial killers that can span into hundreds or thousands of patients, and the longevity into which they conduct their careers which can span decades, it is sufficient to say that the efforts of the medical community to prioritize patient safety in this regard has failed as medical serial killing is *still* a modern and ongoing problem.

This review serves as a call to action on the part of all international healthcare systems to face the harsh and unpalatable reality that the medical field is an opportunistic one in the hands of those with criminal intentions. There is a real need for the implementation of systems that can intercept these persons—all the way from critical hiring practices to M&M meetings, where patient outcomes can be triaged as learning opportunities to those which require further investigations, to standardizations and quality assurance of death certificates that are often the gatekeepers of further investigations by the police. This is also a call on those members of law enforcement to facilitate cooperative interactions with staff and hospitals—rather than a “witch hunt” style of investigations.

Law and Medicine are professions that often collide and conflict—but we wish to reiterate that Law and Medicine are a symbiotic relationship [46, 47]. The Law allows Medicine to conduct itself with great privilege through which physicians may care for with due consideration of all of the morals and ethics and thought that went into such allowances. However, we cannot turn a blind eye when such provisions may be turned on their own head and allow an event, such as the murder of a patient to take place, and moreover turn back doubly on the Law itself which is left with the daunting task to prove guilt beyond a reasonable doubt. There is an area where our true symbiosis maybe seen, and it lies in the root philosophy of Medicine itself—*Primum non Nocere* (Do No Harm). Here is an area of mutual concern which sets the scene for a more collaborative and integrative scenario dedicated to rooting out the most grotesque of outcomes—the murder of a patient.

## Key points

1. Healthcare serial killing involves the intentional death of a patient by any healthcare professional (nurse, doctor, etc.), in any healthcare setting (hospital, clinic, nursing home).
2. Healthcare serial killing is an ongoing and modern problem faced by healthcare systems worldwide; each healthcare system has its own opportunities for malice.
3. Healthcare serial killers are opportunistic as they may conduct themselves in specialties with vulnerable patient groups, at night shifts or staff rounds, and rely on healthcare setting which facilitates their actions by providing the means such as poisons (potassium, insulin, etc.) or tampering of ventilatory equipment.
4. Specific training is needed to increase awareness and promote the management of such cases, as well providing the means to report in a safe and conducive manner when suspicions arise against a suspected person, and collaboration with law enforcement.

## Data Availability

Not applicable.

## Abbreviation

M&M: Morbidity and Mortality
